# Origin of Fat in the Animal Body

**Published:** 1845-04

**Authors:** 


*Origin of Fat in the Animal Body.*—The French chemists continue their experiments upon this important physiological question, being by no means willing to admit that the formation of butyric acid in fermentation from amylaceous principles, is conclusive as regards the origin of fat in the animal body. M. Boussingault has recently published a series of experiments, prefacing his paper with some sarcastic remarks on Dr. Lyon Playfair's communication to the chemical society. He says that Dr. Playfair, in *four* days, tried the influence of four distinct regimens on lactation in the cow, forgetting, in his hurry, to determine the amount of fatty matters in the food, but taking, hypothetically, 1½ per cent. as their contents, whereas there was more than double that amount. Dr. Playfair's experiments Boussingault regards as valueless. In order to throw some light upon this subject, the latter carefully weighed two cows, and subjected them to a rigid diet, analyzing their food, their milk, and excrements.

Boussingault found that the cows, when fed upon beet-root or potatoes, invariably lost weight to a considerable extent, even when they had a superabundant supply. He observes upon this, that an animal's food may be insufficient from various causes. First, if it does not contain a sufficient amount of nitrogenous principles to supply the place of those continually separated from the organism. Secondly. If the amount of carbon be insufficient to replace that burnt or secreted. Thirdly. If there be any deficiency of salts, particularly phosphates. Fourthly. If the food be not sufficiently rich in fatty matters.

The conclusion to which his experiments have led M. Boussingault is, that the food of the herbivora should always contain substances analogous to fat, and which is destined to aid in the production of the fat of the tissues, or in the formation of the several secretions, which contain a considerable proportion of fatty matters, as the bile and milk. If cows continue to yield milk and other secretions, which, in a state of health, are the result of proper feeding, after they are supplied with food wanting in fatty principles, it must be at the expense of their own fat, and they will lose weight daily. A cow, whose food is suddenly changed to aliments deficient in fat, will, perhaps, continue to yield for some days the same weight of butter, but with a loss of fat from the tissues, and the health of the animal will accordingly suffer.

Other experiments on pigs, according to M. Dumas, gave the same results.—*Ib.*

